# Progressive Impairment of Prepubertal Growth in Children With APECED

**DOI:** 10.1210/clinem/dgae209

**Published:** 2024-04-05

**Authors:** Viivi Saari, Venla Alanko, Elina Holopainen, Outi Mäkitie, Saila Laakso

**Affiliations:** Children's Hospital and Paediatric Research Centre, Helsinki University Hospital, Helsinki 00290, Finland; Research Program for Clinical and Molecular Metabolism, University of Helsinki, Helsinki 00290, Finland; Children's Hospital and Paediatric Research Centre, Helsinki University Hospital, Helsinki 00290, Finland; Research Program for Clinical and Molecular Metabolism, University of Helsinki, Helsinki 00290, Finland; Research Program for Clinical and Molecular Metabolism, University of Helsinki, Helsinki 00290, Finland; Department of Obstetrics and Gynaecology, Helsinki University Hospital, Helsinki 00290, Finland; Children's Hospital and Paediatric Research Centre, Helsinki University Hospital, Helsinki 00290, Finland; Research Program for Clinical and Molecular Metabolism, University of Helsinki, Helsinki 00290, Finland; Folkhälsan Research Centre, Biomedicum, Helsinki 00290, Finland; Department of Molecular Medicine and Surgery, Karolinska Institute, Stockholm 171 77, Sweden; Children's Hospital and Paediatric Research Centre, Helsinki University Hospital, Helsinki 00290, Finland; Research Program for Clinical and Molecular Metabolism, University of Helsinki, Helsinki 00290, Finland; Folkhälsan Research Centre, Biomedicum, Helsinki 00290, Finland

**Keywords:** autoimmunity, growth failure, polyendocrinopathies, AIRE, childhood chronic illness

## Abstract

**Context:**

Subjects with autoimmune polyendocrinopathy-candidiasis-ectodermal dystrophy (APECED) have subnormal adult height. There are several potential APECED-related risk factors for suboptimal height attainment during childhood.

**Objective:**

To determine the growth patterns in children with APECED.

**Methods:**

This retrospective longitudinal study included 59 children with APECED from the Finnish national APECED cohort and assessed length/height and weight z-scores from birth to the end of prepuberty.

**Results:**

Collectively, 59 children (30 [51%] girls) were included. Their median birth weight z-score (−0.60) was below the population average; 12 (20%) patients were born small for gestational age. Height attainment progressively declined from birth until the end of prepuberty (z-score −1.95), whereas weight-for-height z-score did not (+0.26). Of the 59 patients, 38 (64%) had all height z-scores below 0 during prepuberty, and 7 (12%) had z-scores below −2.0. Age at the end of prepuberty, number of APECED manifestations, duration of glucocorticoid treatment, and growth hormone deficiency correlated negatively with height z-score at the end of prepuberty (*P* < .0001; *P* = .041; *P* = .013; *P* = .034, respectively).

**Conclusion:**

Children with APECED had a progressive growth impairment from birth through prepuberty. Multiple predisposing risk factors were recognized, including disease severity and growth hormone deficiency. Timely interventions are needed to ensure optimal height attainment and new treatment options need to be developed.

Autoimmune-polyendocrinopathy-candidiasis-ectodermal dystrophy (APECED; OMIM #240300) is a disorder arising from mutations in the autoimmune regulator (*AIRE)* gene (21q22.3) (1). AIRE participates in the development of central tolerance as a transcription regulator in the thymic medullary epithelial cells. Mutations in *AIRE* lead to impaired recognition of autoreactive T-cells, deficiency of regulatory T-cells, and development of autoantibodies against tissue-specific antigens (2). Clinical presentation of APECED is variable and over 20 different manifestations have been described (3). The classical manifestations of APECED are chronic mucocutaneous candidiasis (CMC), hypoparathyroidism (HP), and primary adrenocortical insufficiency (PAI) (4). In addition to endocrinopathies, patients may develop nonendocrine manifestations, such as intestinal dysfunction and hepatitis (3).

Multiple manifestations such as PAI (5), hypothyroidism (6), hypogonadism (7, 8), intestinal dysfunction (9), and their treatments (10) may disturb growth in children with APECED. We have previously shown, that both Finnish female and male individuals with APECED are significantly shorter than their mid-parental target height during puberty and in adulthood, with the average adult height z-score being −1.30 in both women and men (11, 12). However, data on birth size and prepubertal growth in children with APECED are lacking, and it is unknown how the various disease components impact childhood growth.

In this study, we present retrospectively collected data on prepubertal growth in a large cohort of Finnish children with APECED and describe risk factors for growth impairment in children with APECED.

## Methods

### Study Subjects

**Figure 1.**
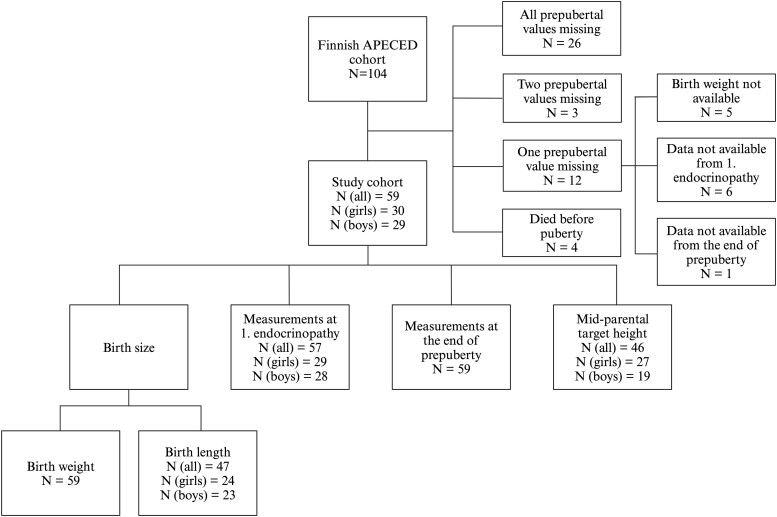
Flowchart of participant selection in the study.

All subjects (N = 104) with APECED were identified from the Finnish APECED cohort (4, 13). Each subject in the cohort met the diagnostic criteria for APECED: 2 of the 3 classical manifestations and/or identified pathogenic variants in *AIRE* (4). In the present study, we included all anthropometric data for those subjects who had anthropometric data available from at least 3 specified time points: birth, time of the first endocrinopathy, and the end of prepuberty. We also included 2 subjects who did not develop any endocrine manifestations during prepuberty but had identified pathogenic variants of *AIRE*. If the time between specified timepoints (the end of prepuberty, time at the first endocrinopathy) and nearest length/height/weight measurement was greater than 6 months, the subjects were not included in the study. The final study cohort included 59 children (59/104, 57%) (Fig. 1). This study was carried out according to the declaration of Helsinki and an ethical approval was obtained from the Research Ethics Committee of the Hospital District of Helsinki.

### Clinical Data

Data were collected from clinical and study records for the years from 1952 to 2021. All length/height and weight measurements were collected and analyzed from birth until the end of prepuberty. The median number of height measurements per subject was 18 (interquartile range [IQR], 11-28). Subjects were followed at least every 3 to 6 months during prepuberty depending on the disease severity. Pubertal development was classified according to Tanner stages (14), as assessed by the pediatric endocrinologist responsible for follow-up. Growth data were collected until the last visit prior to the visit when puberty was observed to have begun (Tanner stage 2 for breast development in female subjects, Tanner stage 2 for genital development [testicular volume ≥ 4 mL] in male subjects) or until pubertal onset was considered delayed (Tanner stage 1 at age 13 years in females, 14 years in males) (15, 16). Birth weight and length z-scores were calculated using Finnish growth references according to pregnancy weeks (17). Birth weight was available for all 59 subjects whereas birth length was available for 47 (80%). Z-scores were calculated according to the exact gestational age in preterm pregnancies and according to 40 + 0 weeks if the pregnancy was full-term (birth between gestational weeks 37 + 0 and 42 + 0). Z-scores for height and weight-for-height were calculated according to Finnish growth references (18). We chose to use weight-for-height instead of body mass index because it has been systematically used in Finland especially for prepubertal children. Mid-parental target height was calculated as the mean of the parents' heights and corrected for sex (−6.8 cm for girls and +6.8 cm for boys) (19). These were converted to z-scores according to the Finnish national growth references (18).

The ages at diagnosis of the following APECED manifestations were collected if they manifested before puberty: CMC, HP, PAI, diabetes, growth hormone deficiency (GHD), hypothyroidism, alopecia, enamel dysplasia, exocrine pancreas insufficiency, gastritis, hepatitis, keratitis, rash with fever, tubulointerstitial nephritis, and vitiligo. In addition, age at diagnosis of primary ovarian insufficiency (POI) in girls and of hypogonadism in boys was determined if developed during puberty before the attainment of adult height. The total duration of glucocorticoid treatment was calculated, including both replacement and treatment indications, if treatment lasted for more than 2 weeks during prepuberty. Data on infections that required antibiotic treatment and/or hospitalization as well as certain viral infections (herpes virus, measles, mumps, rubella, smallpox) were collected.

### Statistical Analysis

All variables are reported as median (IQR). Comparisons between height and weight in different time points were performed with Wilcoxon test. Spearman correlation test was used to calculate the correlation between values (height and glucocorticoid usage, number of manifestations, age at the end of prepuberty, and number of infections; birth year and birth size). The differences between height at the end of prepuberty according to selected manifestations (GHD, HP, PAI, POI) were tested with Mann-Whitney U-test. Significance was set at *P* < .05. Statistical analyses were conducted with GraphPad Prism 9 for macOS (version 9.5.1).

## Results

### Cohort Characteristics and Manifestations of APECED During Prepuberty

Altogether 59 children (30 [51%] girls) were included in the study. Their birth year ranged from 1952 to 2009 (median, 1979). Of these subjects, 57 (97%) were born full-term, and 2 (3%) preterm (gestational weeks 35 + 0 and 36 + 6). The *AIRE* genotype was known for 56 subjects with 42 being homozygous for the Finnish major mutation c.769C>T (p.Arg257Ter). In 12 subjects, the major mutation was compounded with c.967_979del13 (p.Leu323fs) (n = 4), c.1638A>T (p.Ter546Cys) (n = 3), c.932G>A (p.Cys311Tyr) (n = 2), c.891C>A (p.Asp297Glu) (n = 2), and c.1163^1164insA (p.Met388fs) (n = 1). For 2 patients, only one *AIRE* variant had been identified: c.967_979del13 and c.932G>A. The median age at the end of prepuberty was 12.3 years (IQR, 11.2-13.1).

We first analyzed the prevalence and age at onset of endocrine manifestations during prepuberty. The first endocrinopathy was diagnosed at the median age of 6.0 (IQR, 4.7-9.1) years, being HP in 37 subjects (63%), PAI in 17 (29%), GHD in 2 (3.4%), and hypothyroidism in 1 (1.7%). In addition, 2 subjects (3.4%) had not been diagnosed with any endocrine manifestations by the end of prepuberty. In girls, HP was the first endocrinopathy in almost all (28/30, 93%), whereas variability was greater in boys, with PAI being the most common first endocrinopathy (16/29, 55%). By the end of prepuberty, GHD was diagnosed at the median age of 10.7 years in 8 (14%) subjects who all received growth hormone treatment. The median duration of growth hormone treatment before entering puberty was 3.0 years. Brain magnetic resonance imaging results were available for 4 of the 8 children with GHD, and none of them had any significant findings in the region of pituitary gland. Age, height, and weight measurements at the time of different manifestations are shown in Table 1.

**Table 1.** Endocrine manifestations and median (range) age, height, and weight at the time of their diagnosis in 59 subjects with APECED

Manifestation	Girls	Boys	Total
Hypoparathyroidism, N	29	13	42
Age, years	5.58 (2.23-11.1)	6.71 (2.06-11.0)	5.77 (2.06-11.1)
Height, z-score	−1.47 (−4.74 to +1.12)	−0.97 (−2.65 to +0.47)	−1.40 (−4.74 to +1.12)
Weight-for-height, z-score	−0.04 (−2.54 to +1.31)	+0.92 (−1.32 to +3.20)	+0.025 (−2.54 to +3.20)
Primary adrenocortical insufficiency, N	16	25	41
Age, years	7.03 (2.53-11.6)	8.89 (4.23-12.7)	7.96 (2.53-12.7)
Height, z-score	−1.70 (−5.17 to +0.55)	−1.23 (−3.31 to +1.81)	−1.48 (−5.17 to +1.81)
Weight-for-height, z-score	−0.22 (−2.68 to +1.48)	+0.15 (−2.56 to +2.09)	+0.11 (−2.68 to +2.09)
Growth hormone deficiency, N	2	6	8
Age, years	8.83 (5.87-11.8)	10.7 (5.37-13.0)	10.7 (5.37-13.0)
Height, z-score	−3.10 (−3.57 to −2.63)	−2.89 (−3.55 to −1.48)	−2.89 (−3.57 to −1.48)
Weight-for-height, z-score	−0.13 (−0.86 to +0.60)	+0.52 (0.006 to +2.09)	+0.46 (−0.86 to +2.09)
Diabetes, N	2	2	4
Age, years	10.2 (9.35-11.0)	6.61 (3.97-9.25)	9.30 (3.97-11.0)
Height, z-score	−2.54 (−2.85 to −2.23)	−1.35 (−3.08 to +0.37)	−2.54 (−3.08 to +0.37)
Weight-for-height, z-score	−0.38 (−0.62 to −0.13)	−1.09 (−1.61 to −0.57)	−0.59 (−1.61 to −0.13)
Hypothyroidism, N	0	4	4
Age, years	—	7.00 (4.74-13.8)	7.00 (4.74-13.8)
Height, z-score	—	−1.52 (−3.77 to −0.10)	−1.52 (−3.77 to −0.10)
Weight-for-height, z-score	—	+0.94 (0.39 to +1.58)	+0.94 (0.39 to +1.58)

### Target Height and Birth Size

**Figure 2.**
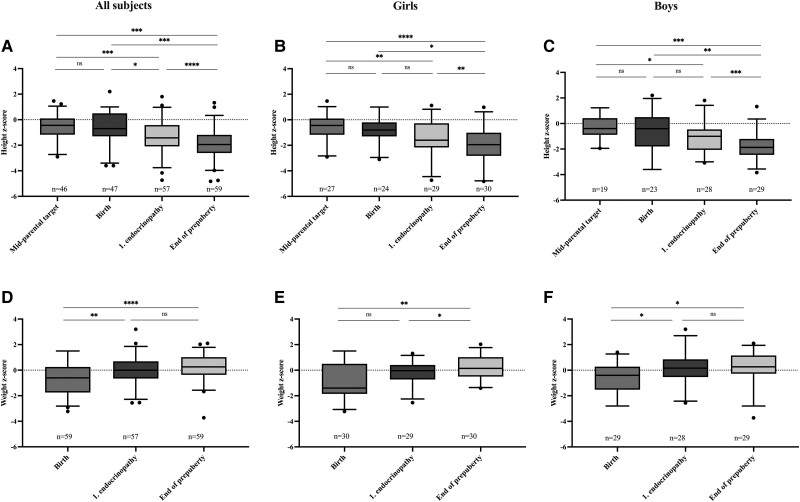
Comparison of median length/height (A-C) and median weight/weight-for-height z-scores (D-F) (box indicating 25th to 75th percentile, whiskers 5th to 95th percentile) during different phases of prepubertal development in 59 subjects (30 girls, 29 boys) with APECED. * = *P* < .05; ** = *P* < .005; *** = *P* < .001; **** = *P* < .0001; ns = non-significant.

The median mid-parental target height z-score was −0.45 for 46 subjects (27 girls, 19 boys). It was in girls −0.45 corresponding to 164.7 cm, and in boys −0.39 corresponding to 178.3 cm. The median birth weight z-score was −0.60 in all subjects, −1.40 (3055 g; IQR, 2860-3780 g) in girls, and −0.40 (3600 g, 3050-3880 g) in boys (Fig. 2D-2F). Altogether, 12 subjects (20%) were small for gestational age (SGA, birth weight z-score adjusted for gestational age < −2.0). Birth length z-score was below that in the average Finnish population being −0.70 in the whole cohort, −0.80 (49.0 cm) for girls, and −0.40 (50.5 cm) for boys, but it did not significantly differ from mid-parental target height (Fig. 2A-2C). The year of birth did not correlate with birth weight z-scores (*r* = −0.12; 95% CI [−0.37 to +0.15], *P* = 0.35).

### Prepubertal Height and Weight Gain

**Figure 3.**
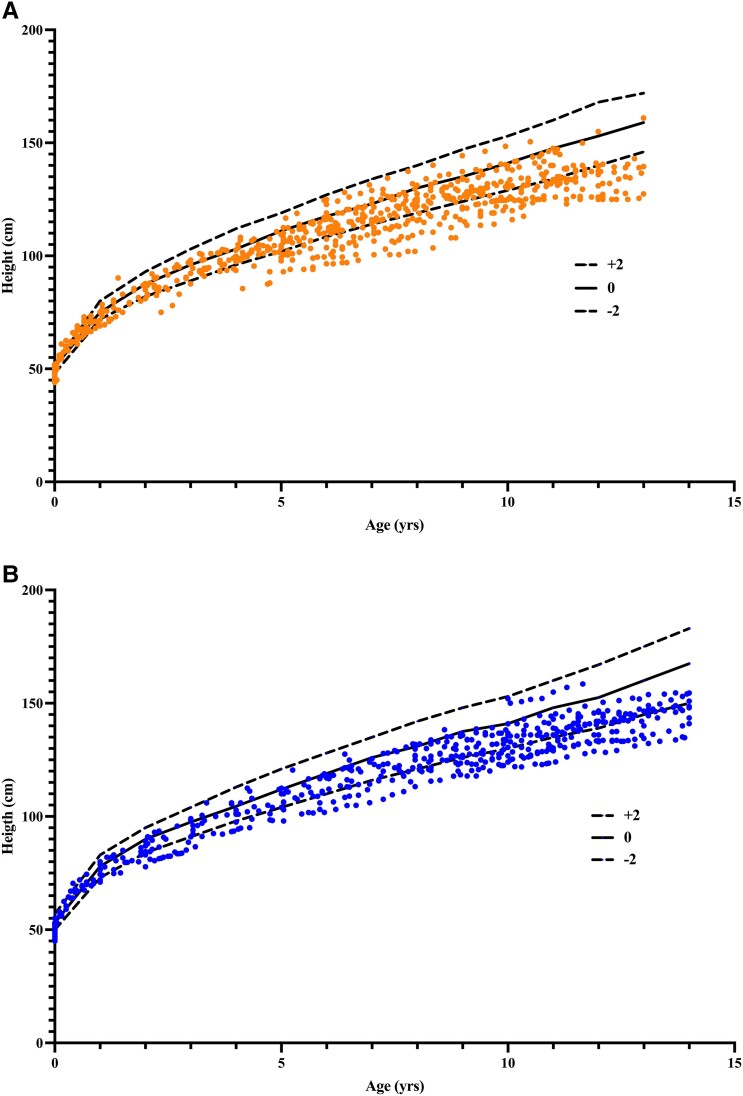
All length and height measurements from birth until the end of prepuberty in 59 subjects with APECED compared to Finnish national growth curves of z-score −2, 0, and +2 in girls (A); and boys (B).

All height measurements from girls and boys were compared with the Finnish growth curves (Fig. 3). Of the 30 girls with APECED, 20 (67%) had height z-score below normal mean and 5 (17%) below −2.0 during the entire prepuberty. Of the 29 boys, 18 (62%) grew below the normal mean and 2 (6.9%) below −2.0. Regarding the growth during the first years, median height z-score was at the age of 1 year −1.16 (n = 21, IQR −2.0 to −0.18) and at the age of 2 years, −0.80 (n = 27, −1.88 to −0.16).

To analyze longitudinal growth patterns in the cohort, we compared height z-scores between 3 time points (birth, first endocrinopathy, and the end of prepuberty) and in relation to mid-parental target height (Fig. 2A-2C). The median height z-score declined from birth to the time of first endocrinopathy, to −1.42 in all subjects, −1.60 in girls, and −1.00 in boys. The decline continued from the first endocrinopathy to the end of prepuberty, when the median height z-score was −1.95 in all subjects, −1.95 in girls, and −1.87 in boys. In the whole cohort, height z-scores differed significantly (*P* < .05) between all 3 time points. Heights at the time of first endocrinopathy and at the end of prepuberty differed also significantly from mid-parental target height in the whole cohort and in both sexes separately. Information regarding final adult height was available for altogether 48 subjects. Their median height z-score was −1.31 (IQR, −2.19 to −0.77) and significantly below their mid-parental target height (*P* ≤ .0001).

The children born SGA had experienced catch-up growth by the time of diagnosis of the first endocrinopathy, with height z-scores not significantly different from those born with birth size appropriate for gestational age (children with SGA [n = 11] vs others [n = 46]; median [IQR]; −1.62 [−3.53 to −0.50] vs −1.27 [−1.92 to −0.37], *P* = .16). However, 5 of them still had a height z-score below −2.0 when the first endocrinopathy was diagnosed at the median age of 6.4 years. The height z-score was above 0 for the whole prepuberty in 2 subjects: one of them had 5 manifestations (HP and PAI diagnosed at the age of 2.5 years, and CMC, enamel dysplasia, and rash with fever); and the other had 2 manifestations (PAI diagnosed at the age of 10.0 years, and CMC).

Next, we determined the weight gain during prepuberty. Z-scores for weight-for-height (height-adjusted weight) were calculated for 2 time points: at the time of the first endocrinopathy and at the end of prepuberty (Fig. 2D-2F). The median weight-for-height z-score at the time of the first endocrinopathy was −0.004 in the whole cohort, −0.036 in girls, and +0.16 in boys. An increase was seen in all groups by the end of prepuberty, median z-score being +0.26 in the whole cohort, +0.13 in girls, and +0.26 in boys. Birth weight z-score differed significantly from the height-adjusted weight z-score at the end of the prepuberty in the whole cohort (*P* < .0001) and in girls and boys separately (*P* = .0023; *P* = .016). Three subjects (5.3%) were underweight (weight-for-height z-score < −2.0) at the time of first endocrinopathy and one (1.7%) at the end of prepuberty.

### Correlations Between Height and Clinical Characteristics

**Figure 4.**
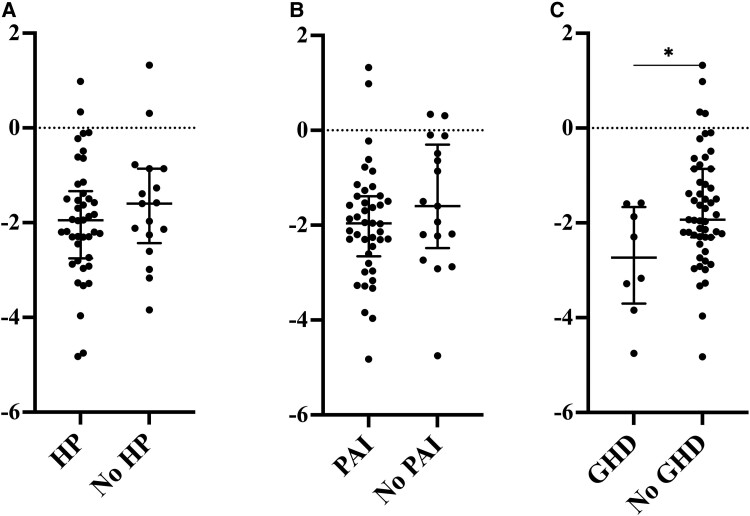
Comparison in median height z-score (IQR) at the end of prepuberty in 59 APECED subjects in relation to different manifestations seen during prepuberty. A, Hypoparathyroidism (HP); B, Primary adrenocortical insufficiency (PAI); C, Growth hormone deficiency (GHD). * = *P* < .05.

We evaluated how the most common endocrine manifestations associated with height attainment. Height z-scores at the end of prepuberty according to the presence of these manifestations are shown in Fig. 4. Subjects with HP (42/59) or PAI (42/59) did not have significantly lower height z-score at the end of prepuberty (*P* = .45; *P* = .20) than those without HP or PAI. In contrast, GHD associated with lower height z-score at the end of prepuberty, median height z-score being −2.73 for those with GHD and −1.93 for those without GHD (*P* = .034). To explore whether GHD alone explained the growth decline in longitudinal analysis between birth and the end of prepuberty, we excluded the 8 subjects with GHD from the analysis. By this, the difference in height z-score between birth and the first endocrinopathy became insignificant in the whole cohort (*P* = .14), but the length/height z-score differences between birth and the end of prepuberty as well as the onset of first endocrinopathy and the end of prepuberty remained statistically significant (*P* = .008 and *P* < .0001, respectively).

**Figure 5.**
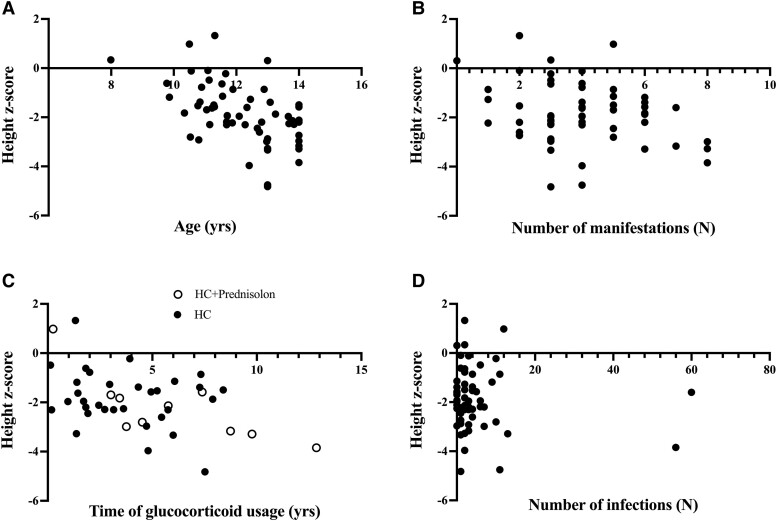
Correlation between the height z-score at the end of prepuberty and age (A); duration of glucocorticoid treatment (B); the number of manifestations (C); and the number of infections in 59 subjects with APECED (D). Abbreviation: HC, hydrocortisone.

We then analyzed correlations between the height z-score at the end of puberty and age, number of manifestations, duration of glucocorticoid treatment, or number of infections (Fig. 5). Age at the end of prepuberty correlated negatively with height z-scores at the end of prepuberty, reflecting effects of delayed onset of puberty on height (*r* = −0.52 [−0.69 to −0.30], *P* < .0001). However, there was a positive correlation between actual height (cm) and age at the end of prepuberty (*r* = +0.40 [+0.16 to +0.60], *P* = .002).

As an indicator of disease severity, we calculated the total number of manifestations at the end of prepuberty. This correlated negatively with height z-score at the end of prepuberty (*r* = −0.27 [−0.49 to −0.011], *P* = .041). The median number of manifestations diagnosed during prepuberty was 4.0 (IQR, 3-5). As supraphysiological glucocorticoid treatment may delay growth, we correlated duration of glucocorticoid treatment with height z-score at the end of prepuberty. Glucocorticoids were used by 42 subjects (71%): all 42 used a hydrocortisone substitution for PAI. In addition, 10 of them were treated with prednisolone for hepatitis (n = 7), rash with fever (n = 3), tubulointerstitial nephritis (n = 1), and/or uveitis (n = 1). The daily dose of hydrocortisone varied between 3.75 and 20.0 mg, and the daily dose of prednisolone varied between 1.25 and 50 mg. The median duration of glucocorticoid usage was 3.8 years [IQR, 1.82-6.02]. Significant correlation between height at the end of prepuberty and duration of glucocorticoid treatment was found only in patients using both hydrocortisone replacement and treatment doses of prednisolone (*r* = −0.77, [−0.94 to −0.27], *P* = .013), but not in patients receiving only hydrocortisone replacement (*r* = −0.15 [−0.48 to +0.22], *P* = .42, Fig. 5). Further, the number of infections during prepuberty did not correlate with height z-score at the end of prepuberty (*r* = −0.046 [−0.31 to +0.22], *P* = .74). The median number of infections during prepuberty was 2 (IQR, 1-6).

Finally, we wanted to assess if later development of hypogonadism was associated with preceding alterations in growth during prepuberty. The girls were divided into 2 groups: girls who developed POI before attaining adult height (n = 15) and girls who had a normal ovarian function at the time of adult height attainment (n = 13). The median height z-score at the end of prepuberty was −2.19 in girls who developed POI and −1.50 in girls who did not develop POI before the attainment of adult height (*P* = .24). In boys, hypogonadism was only diagnosed in 3 patients before the attainment of adult height and therefore such comparison could not be done.

## Discussion

We analyzed retrospectively prepubertal growth in the 59 children with APECED. Both median birth weight and length were smaller in children with APECED compared to average Finnish children. Height attainment declined, whereas weight-for-height did not decrease during prepuberty. The number of APECED manifestations, duration of glucocorticoid treatment, and GHD correlated negatively with the height z-score at the end of prepuberty.

The data presented here are unique, as birth measurements or prepubertal growth in children with APECED have not been described before. Even data from animal studies are scarce. A previous report on an *Aire* knock-out rat model showed that Aire deficiency did not affect growth during the first 20 weeks of life (20). Our findings are in contrast with this, since even the median birth weight and birth length z-scores were below the normal average in both girls and boys with APECED. It is not known how *AIRE* mutations could affect growth already during the fetal period. One possible explanation for small birth size is the dysfunction of the thymus. The thymus is very active during the fetal period and participates in protection against inflammation and infections (21). The role of *AIRE* in the thymus is fundamental. One human study has shown a correlation between the smaller size of thymus and smaller birth weight, whereas, in another study, no such correlation was found (22, 23). The size of thymus at birth or perinatal infections was not systematically studied in our cohort. This novel aspect of APECED requires further studies. The data on the correlation between mid-parental target height and birth length are scarce but in one recent study, mid-parental height was identified as a strong determinant of both intrauterine growth and birth size (24).

Several different manifestations of APECED can cause disturbances in prepubertal height attainment. Data on the effects of autoimmune HP on longitudinal growth are scarce but suggest that HP may impair growth. In a case study on a family with isolated primary HP, subjects were shorter than average at the age of 12 to 13 years (25). In another case study, one person who experienced total parathyroidectomy at age 30 weeks due to neonatal severe hyperparathyroidism, was followed for 20 years. Although he had adequate calcium and later parathyroid hormone substitution, his adult height z-score was −1.7 (26). In our data, subjects with HP were not significantly shorter than those without. However, HP was the most common manifestation seen in our cohort, diagnosed in 71% of the subjects. The second most common manifestation seen was PAI, diagnosed in 69% of the subjects. Excess of glucocorticoids may impair growth in several different ways: general catabolic influences, interference of the GH-IGF-1-axis, and suppression of several genes in the growth plate (27). Children with autoimmune PAI are shorter than their peers at the time of diagnosis, but with glucocorticoid and mineralocorticoid replacement, their growth rate was considered normal. However, their adult height has been reported to be shorter than their mid-parental target height (5). In our study, we did see a significant correlation between the duration of glucocorticoid treatment and height z-score at the end of prepuberty only in those patients who had received prednisolone treatment for another manifestation in addition to hydrocortisone replacement for PAI. However, the causality of the association cannot be evaluated retrospectively. The median weight-for-height z-score was slightly above average at the time of PAI diagnosis, which may reflect early stages of adrenal insufficiency. GHD was the only manifestation that alone associated with a statistically significant difference in height at the end of prepuberty. However, data analysis without subjects with GHD indicated, that GHD was not the only factor with a negative effect on growth. Growth hormone replacement therapy usually allows accelerated high velocity and obtainment of target adult height (28). It should be noted that treatment for GHD has only been available from the 1980s and therefore GHD has not been diagnosed or treated in the older subjects included in the study.

As delayed puberty causes delayed longitudinal growth (29), we excluded height measurements after the age of 13 in girls and 14 in boys because their pubertal development was considered delayed (15, 16). Our data showed that the subjects' age at the end of prepuberty and height z-score correlated negatively whereas actual height correlated positively with age. This showed that the growth of subjects who started pubertal development later did not halt at any point but continued with prepubertal growth velocity whereas peers had entered puberty with a faster velocity. We also investigated whether development of POI after prepuberty impacted height gain already before puberty. Development of POI is a gradual process, and the depletion of the ovarian reserves can continue for years before the diagnosis is reached (30). Adequate concentrations of estrogen are needed for normal longitudinal growth (7). We previously showed that females diagnosed with POI before attainment of adult height were shorter at the time of menarche, but their adult height did not differ from those with normal ovarian function (11). Therefore, we hypothesized that girls who developed POI before reaching adult height might be shorter even at the time of prepuberty because of the gradual impairment in estrogen production and depletion of ovarian reserve already in prepuberty. Indeed, the girls with subsequent development of POI tended to be shorter already in prepuberty than those who did not develop POI, but the difference did not reach statistical significance.

In addition to these endocrine manifestations, other disease manifestations in APECED can also affect longitudinal growth, including intestinal dysfunction (9) and exocrine pancreatic insufficiency (31). In the American APECED cohort, intestinal dysfunction was diagnosed in 77% of subjects aged under 15 years (9). In intestinal dysfunction, absorption of nutrients can be impaired and lead to malnutrition and poor weight gain. In our retrospective study, we could not assess reliably the prevalence or severity of intestinal dysfunction. Because the weight of our subjects was normal in relation to height, it seems plausible that severe intestinal dysfunction was not common, and absorption of nutrients was sufficient in most of them to sustain normal weight gain. However, absorption of certain nutrients could still have been inadequate.

Recurrent infections can disturb normal growth. We collected the data on infections treated during prepuberty to determine whether children with APECED had more infections than the average population and if this affected their growth. The number of infections varied greatly during the prepubertal time but did not correlate with the height at the end of prepuberty. CMC, a common APECED manifestation, can cause painful sores inside oral mucosa making eating difficult and therefore causing malnutrition (32). The retrospective nature of our study prevented us from reliably assessing the severity of CMC in our series. However, since our subjects had normal weight-for-height z-scores on average, it seems unlikely that severe CMC would play a major role.

There are certain limitations in our study. The number of subjects in our study is relatively small, and the disease phenotype varies significantly within the cohort. However, this is still the largest study on childhood growth in APECED and involves a carefully monitored cohort with longitudinal growth data from birth to puberty for each included individual. The retrospective nature also limited the study and prevented detailed assessment of some disease components and timepoints such as age of 1 and 2 years that may be more prognostic for adult height than birth size. To further investigate the pathogenesis behind linear growth disturbances during the fetal period and prepuberty, prospective studies are needed. However, in the case of rare diseases such as APECED, establishing the appropriate study design to accommodate this limitation may not be feasible.

In conclusion, our study showed that children with APECED have a growth impairment already prenatally, they are shorter than average children already at the time of birth, and this difference increases during prepuberty. Our results indicate that diagnosis of GHD, age at the end of prepuberty, duration of glucocorticoid treatment, and number of manifestations correlated negatively with height at the end of prepuberty, whereas HP, PAI, and number of infections did not. Therefore, careful follow-up and timely interventions are warranted to ensure optimal growth during prepuberty. Furthermore, more research is needed to explore factors affecting prenatal growth in subjects with APECED and to develop novel therapeutic approaches addressing the autoimmune mechanisms behind APECED.

## Data Availability

Restrictions apply to the availability of data generated or analyzed during this study to preserve patient confidentiality. The corresponding author will on request detail the restrictions and any conditions under which access to some data may be provided.

## Abbreviations

AIRE: autoimmune regulator
APECED: autoimmune polyendocrinopathy-candidiasis-ectodermal dystrophy
CMC: chronic mucocutaneous candidiasis
GHD: growth hormone deficiency
HP: hypoparathyroidism
IQR: interquartile range
PAI: primary adrenocortical insufficiency
POI: primary ovarian insufficiency
SGA: small for gestational age
